# Mechanical Properties of Lumbar and Cervical Paravertebral Muscles in Patients with Axial Spondyloarthritis: A Case-Control Study

**DOI:** 10.3390/diagnostics11091662

**Published:** 2021-09-10

**Authors:** Juan L. Garrido-Castro, I. Concepción Aranda-Valera, José Peña-Amaro, Alfonso Martínez-Galisteo, Cristina González-Navas, Daiana P. Rodrigues-de-Souza, Sandra Alcaraz-Clariana, Lourdes García-Luque, Iago R. Martínez Sánchez, Clementina López-Medina, Eduardo Collantes-Estévez, Francisco Alburquerque-Sendín

**Affiliations:** 1 Maimonides Biomedical Research Institute of Cordoba (IMIBIC), 14004 Cordoba, Spain; cc0juanl@uco.es (J.L.G.-C.); conchita.87.8@gmail.com (I.C.A.-V.); cm1peamj@uco.es (J.P.-A.); crsgonzaleznavas@yahoo.es (C.G.-N.); clementinalopezmedina@gmail.com (C.L.-M.); eduardo.collantes.sspa@juntadeandalucia.es (E.C.-E.); falburquerque@uco.es (F.A.-S.); 2 Department of Rheumatology, University Hospital Reina Sofía, 14004 Cordoba, Spain; 3 Department of Medical and Surgical Sciences, University of Cordoba, 14004 Cordoba, Spain; 4 Department of Morphological and Social Health Sciences, University of Cordoba, 14004 Cordoba, Spain; 5 Faculty of Veterinary, University of Cordoba, 14014 Cordoba, Spain; an1magaa@uco.es; 6 Department of Nursing, Pharmacology and Physical Therapy, University of Cordoba, 14004 Cordoba, Spain; m72alcls@uco.es (S.A.-C.); z12galul@uco.es (L.G.-L.); 7 Advanced Informatics Research Group (GIIA) TIC-252, University of Cordoba, 14014 Cordoba, Spain; iamarsan1460@gmail.com

**Keywords:** axial spondyloarthritis, cervical muscles, lumbar muscles, muscle tonus, rehabilitation

## Abstract

Background: Axial spondyloarthritis (axSpA) affects spinal muscles, due to inflammation and structural damage. The mechanical properties of the muscles, such as tone or stiffness, could be altered in axSpA. The aim of this work is to analyze the mechanical properties of cervical and lumbar spine muscles in axSpA patients and their relationship with metrology measures, function, disease activity, structural damage and quality of life. Methods: axSpA patients and age/gender/BMI matched healthy controls were recruited. The muscle mechanical properties (MMPs), such as tone or frequency, stiffness, decrement (linear elastic properties), relaxation and creep (viscoelastic properties), of cervical (semispinalis capitis) and lumbar (erector spinae) muscles were bilaterally measured at rest using myotonometry. Additionally, conventional metrology, BASMI (metrology index), BASDAI (disease activity index), mSASSS (radiological structural damage index) and SF-12 (health-related quality of life questionnaire) were used in the axSpA group. Between-groups comparison, intra-group correlations and multivariable regression analyses were performed to achieve the study aims. Results: Thirty-four axSpA patients (mean age: 46.21 ± 8.53 y) and 34 healthy volunteers (mean age: 43.97 ± 8.49 y) were recruited. Both in cervical and lumbar spine, linear elastic parameters were significantly higher in axSpA patients in comparison with controls, while viscoelastic parameters were significantly lower. Lumbar muscle frequency, stiffness, relaxation, creep and cervical muscle elasticity were fair to strongly correlated (|0.346| < r < |0.774|) with age, functional status, activity of disease, structural damage and quality of life in axSpA patients. Furthermore, moderate to good fitted multivariate models (0.328 < R^2^ < 0.697) were obtained combining age, conventional metrology, activity of the disease and function for the estimation of cervical and lumbar MMPs. Conclusion: Mechanical properties of spinal muscles of axSpA patients differ from controls. Lumbar and cervical muscles exhibit greater linear elastic properties and lower viscoelastic properties, which are related with age, clinical and psychophysiological features of axSpA.

## 1. Introduction

Axial spondyloarthritis (axSpA) is a chronic inflammatory rheumatic disease with a high phenotypic heterogeneity. It is characterized by the presence of ‘inflammatory low back pain’, sacroiliitis and the formation of syndesmophytes that can lead to reduced range of motion or even axial ankylosis, together with impaired function, considerable disability and a poor quality of life [1]. The disease may be the result of a complex interplay among genetic risk factors, microbial triggers, inflammation of bone marrow and enthesial structures and new bone formation [2]. Despite the progress made in recent years in the study of factors involved in the development of the disease, the pathophysiology of axSpA is not fully understood [3].

Recently, several authors have hypothesized that certain biomechanical factors, such as axial myofascial hypertonicity, might interact with metabolic and immunological pathways that play a role in the initiation and progression of axSpA [4]. Preliminary studies suggest that muscle tone and stiffness are greater in patients with ankylosing spondylitis (AS) than in age- and sex-comparable control subjects [5]. However, muscle mechanical features, including their elastic and viscoelastic properties, have not been fully characterized in patients with axSpA. Furthermore, the passive resting muscle tone mechanism and its clinical consequences are not completely understood [5], and more information is necessary for a better assessment of this physical property of the spine muscles and its possible relation to the mechanism in the onset and clinical presentation of axSpA [4,6].

In addition, the precise relation between lumbar and cervical muscle biomechanics and other disease domains, such as pain, physical function, disease activity, spinal mobility or quality of life remains unknown [7]. A better understanding of this relationship might help clarify the pathophysiology and improve the quality of assessment of the disease, with clinical implications for the optimization of medications, exercises or physiotherapy used; this is to eventually lead to an improved quality of life of patients with axSpA.

Different approaches have been applied to know the muscle state in spinal disorders, with some of them being subjective, such as palpation, and others difficult to apply and interpret in a clinical setting, such as electromyography (EMG) [7,8]. In other words, specific tools to assess the muscle state of axSpA and, specifically, their mechanical properties, with clinical availability, are scarce [9]. In this sense, MyotonPro© is a handheld device used to measure muscle mechanical properties in different research and clinical fields, including sports performance [10], stroke patients [11], polymeric gel-based tissue phantoms [12] and Parkinson’s disease [13]. The device has recently shown good results in characterizing the paravertebral mechanical features when applied to asymptomatic [14] patients with cervical and lumbar spinal pain [15]. Through the application of mechanical stimuli, this device allows one to measure: (a) tone or state of tension, as the natural oscillation frequency; (b) biomechanical properties, such as dynamic stiffness and logarithmic decrement of natural oscillation, which is inverse to the overall elasticity of the tissue; (c) viscoelastic properties, such as mechanical stress relaxation time and the ratio of deformation and relaxation time, characterizing creep (Deborah number). [16]. MyotonPro has shown good validity [17,18,19] and test-retest reliability [14,18,20].

Thus, the primary objective of this study was to describe cervical and lumbar mechanical properties of spinal muscles in axSpA patients when compared to matched healthy subjects. The secondary aim was to identify associations between the mechanical properties and clinical status.

## 2. Materials and Methods

This is a case-control study conducted between January 1st and September 30th, 2018. A non-probabilistic sampling of consecutive cases was performed among the axSpA patients from daily clinical practice at the rheumatology department of the Hospital Universitario Reina Sofía, Córdoba (Spain). The patients were participants from the CASTRO (Córdoba Axial Spondyloarhritis Task force, Registry and Outcomes Spondyloarthritis Registry) cohort.

### 2.1. Participants

Study inclusion criteria were the following: (1) 18–80 years of age, (2) confirmed diagnosis according to the assessment of spondyloarthritis international society (ASAS) criteria for axSpA [21]. Study exclusion criteria were the following: (1) any type of spinal pain at the time of the study, (2) severe restriction in hip mobility (less than 20° of rotation in either hip), (3) history of vertebral, pelvic or hip fracture, (4) history of spinal surgery, (5) moderate to severe scoliosis (Cobb angle >20°) and (6) pregnancy.

Healthy controls matched by sex, age (±3 years) and body mass index (BMI) (±2 Kg/m^2^) were recruited through advertising among university and hospital workers. Control subjects were excluded if they presented with any of the exclusion criteria for the axSpA group or had any pain at the time of the interview, any neurological or musculoskeletal condition or had received previous spinal surgery. The study protocol was approved by the Hospital Universitario Reina Sofía Hospital’s ethics board (approval number: 0887-N-17). All participants signed an informed consent form.

### 2.2. Clinical Variables

Eligible participants were scheduled for an initial review visit and to sign consent forms, fill out baseline questionnaires and be screened by the study participating physicians. All the following clinical variables were collected by one physician, a rheumatologist with 10 years of clinical experience (I.C.A.-V.). In the axSpA group, measurements for conventional metrology were obtained according to Sieper et al. review [22]: (1) cervical rotation, assessed with a goniometer in sitting position; (2) tragus-wall distance; (3) lateral spinal flexion; (4) modified Schöber test; (5) intermalleolar distance, assessed with a tape measure. The Bath Ankylosing Spondylitis Metrology Index (BASMI) index was also calculated, and a visual analogic scale was used to assess general pain.

Participants also completed self-reported questionnaires for function assessment (Bath Ankylosing Spondylitis Function Index (BASFI), with a range from 0 to 10) and disease activity (Bath Ankylosing Spondylitis Disease Activity Index (BASDAI), with a range from 0 to 10). Radiographic structural damage was scored in axSpA patients according to the modified Stoke Ankylosing Spondylitis Spinal Score (mSASSS) index, which ranges from 0 to 72 using x-ray images [22]. The 12-item Short-Form Health Survey (SF-12) was used to assess the health-related quality of life, divided by the physical component (PCS-12) and the mental component (MCS-12) [23,24]. Each of the 12 items has the possibility of 3 to 5 answers, with lower values indicating poorer health-related quality of life on a scale from 0 to 100. The Spanish versions of all questionnaires were applied, which have shown good clinical metrics and are commonly used in clinical practice. All the measurements were conducted by rheumatologists and physiotherapists experienced in metrology (at least 5 years) at the movement analysis laboratory in our hospital.

### 2.3. Myotonometry Measurements

MyotonPro© (Myoton AS, Tallinn, Estonia) device provides measurements of five muscle mechanical properties (MMPs), such as tone or frequency (Hz), dynamic stiffness (N/m), decrement, creep (Deborah number) and relaxation time of stress (ms). The device applies, by a probe, a controlled pre-load of 0.18 N for an initial compression of the subcutaneous tissue before imposing an additional 15 ms impulse and 0.40 N of mechanical force, which induces a dampened natural oscillation of the tissue. The peak acceleration of the natural oscillation is detected and measured using an accelerometer located at the tip of the probe.

**Figure 1:**
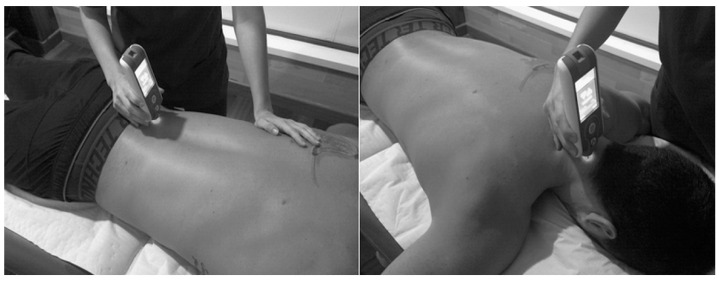
Procedure for measuring lumbar (**left**) and cervical (**right**) muscles using MyotonPro©.

The patient was placed in prone position for 2 min, with the arms by the sides of the body on a physiotherapy stretcher. The measurements points identified by the therapist according to a visual-palpatory test were the erector spinae at 2.5 cm to the right and left of L5, and the semispinalis capitis muscle on the right and left at the level of C4. The testing probe of the myotonometer was placed vertically on the skin surface of the belly of the tested muscle (Figure 1). The muscle measurements were first taken on the left lumbar side, then on the right lumbar side and the process was repeated; then, on the left cervical side and finally on the right side and the process was repeated. All measurements were taken after 10.00 am in the morning to avoid morning stiffness. For our analysis, we used the mean values of two tests (initial and 5 min later) from both sides of the muscles (left and right) for each individual. The same rheumatologist (I.C.A.-V.) that assessed metrology applied the myotonometric evaluation.

The evaluation was repeated 5 min later following the same process for the first 10 subjects of both groups. The test-retest reliability of these measurements was calculated with the first 10 subjects of both groups. Thus, the ICC between the two consecutive measurements produced highly reliable results: from 0.91 to 0.98 for cervical measurements and from 0.97 to 0.98 for lumbar measurements. Standard error of measurement (SEM: cervical < 3%, lumbar < 2%) and minimum detectable change (MDC: cervical < 7%, lumbar < 2%) were also low.

### 2.4. Statistical Analysis

Sample size requirements: Our aim was to achieve a power of 80.0% to detect differences by means of a bilateral unpaired Student’s t-test, taking into account that the level of significance is 5.0%, and assuming that the minimum detectable difference between means is 52.1 N/m and the standard deviation of both groups is 75.0 N/m (according to a pilot study) in the stiffness of the lumbar spinal musculature. Therefore, it would be necessary to include 34 experimental units in the control group and 34 units in the axSpA group. To identify intra-group correlation indexes of 0.5 between the mechanical properties, sociodemographic and clinical variables, with a power of 80% and an alpha of 0.05, at least 30 subjects were necessary. For regression analysis, estimating an average effect size f = 0.25, an alpha level 0.05, power of 0.90 and 2 predictors, the required sample size was 54 subjects. Assuming a data loss of 10%, the axSpA group sample was expanded to 60 patients (G*Power 3.1)

Frequencies, percentages, means, standard deviations and 95% confidence intervals were calculated to describe variables. All numerical variables were considered normally distributed according to Kolmogorov–Smirnov test (*p* > 0.05) and visual evaluation of histograms. Since no significant differences were found between the mechanical properties’ measurements from the left and right sides of the muscles according to paired Student t-tests, the mean of both sides was used for analyses.

For between-group comparison purposes, unpaired Student t-tests were used to identify differences in mechanical properties of cervical and lumbar musculature and in mobility measurements.

To assess intra-group relations between mechanical properties and sociodemographic and clinical features of the axSpA group, Pearson’s correlation coefficient was used to measure the degree of co-variation between the variables obtained by myotonometry and other variables analyzed in patients with axSpA. Correlations were classified as negligible (0.0–0.19), fair (0.20–0.39), moderate (0.40–0.69), strong (0.70–0.89) or almost perfect (0.90–1.00) [25].

Once the sample size was expanded to 60 patients, those variables associated with MMPs were included in a multiple linear regression model through applying the stepwise method, to estimate which sociodemographic and clinical variables predicted stiffness and decrement of the lumbar and cervical muscle tissues separately. The Pearson correlation coefficient was also applied to identify multicollinearity between the variables (defined as *r* > 0.80). To identify the explained variance of each biomechanical properties, a hierarchical regression analysis was conducted. The significance criterion of the critical F value for entry into the regression equation was set at *p* < 0.05. All changes after each step of the model in R^2^ were reported.

All contrasts were bilateral and *p* < 0.05 was considered significant. Statistical analysis was performed using the IBM SPSS Statistics version 25 (SPSS Inc., Chicago, IL, USA).

## 3. Results

### 3.1. Between-Groups Comparisons

Thirty-four patients with axSpA and 34 healthy controls were recruited. Table 1 shows the demographic data of axSpA patients and controls. There were no significant differences between groups either in age, sex, weight or in BMI. Both groups showed overweight values according to BMI. axSpA patients showed significantly lower values in all metrology measurements with the exception of the Schöber test (Table 1).

Table 2 shows results obtained for mechanical properties of cervical and lumbar muscles measured in patients and healthy controls. Values of frequency and stiffness are higher in axSpA with significant differences. Conversely, relaxation and creep are lower in axSpA with even greater differences, especially in lumbar spine. Decrement did not show significant differences in both regions.

### 3.2. Intra-Group Correlations in the axSpA Patients

The pattern of correlations was different depending on the region analyzed. Thus, for the cervical region, the decrement was moderately correlated with the age, BMI, tragus to wall distance, BASMI and mSASSS in a direct sense (0.426 < r < 0.676), and with Schöber test, cervical rotation and chest expansion in an inverse sense (−467 < r < −0.562). Frequency and stiffness were the only other mechanical properties that showed any significant correlation (*p* < 0.05) in this case with age in a direct sense (Table 3).

On the contrary, more significant correlations were identified for the lumbar muscles, in this case being the frequency, stiffness, relaxation and creep, with the mechanical properties involved in the pattern. Thus, these properties were moderate correlated (|0.404| < *r* < |0.697|) with age, tragus to wall distance, BASMI, BASFI (in these cases, the higher the frequency and stiffness and the lower relaxation and creep, the higher age, tragus to wall distance, BASMI and BASFI) and with lateral flexion and Schöber test (in these cases, the higher the frequency and stiffness and the lower relaxation and creep, the lower lateral flexion, Schöber test). Further, moderate to high correlations were identified with mSASSS (|0.489| < *r* < |0.774|), and fair to moderate with pain (in these cases, the higher the frequency and stiffness and the lower relaxation and creep, the higher mSASSS and pain) and MCS-12 (in this case, the higher frequency and stiffness and the lower relaxation and creep, the lower MCS-12) (|0.346| < *r* < |0.444|). Finally, frequency (*r* = −0.489) and stiffness (*r* = −0.493) were moderate and inversely correlated with cervical rotation, and frequency was fair and directly correlated with BASDAI. No correlation was detected between disease duration and any mechanical property in both regions.

### 3.3. Multivariate Regression in the axSpA Group

The regression analysis showed that cervical muscle frequency, characterizing tone, could be partially explained (R^2^ = 0.422) by the combination of age, tragus-wall and lateral flexion (the higher the age, the higher the frequency; the lower tragus-wall distance and lateral flexion, the higher the frequency). For the stiffness, the model achieved a R^2^ of 0.328 when age and cervical rotation were combined, where higher values of both variables were associated with higher values of stiffness. For the decrement, as an inverse measure of the elasticity, the model showed that the age, BASDAI and BASMI were inversely related to the elasticity. Their combination showed a R^2^ of 0.615 (Table S1). No model was identified for cervical muscle relaxation and creep.

For the lumbar region, the frequency was directly related with the intermalleolar distance, age and BASDAI, and inversely related with the modified Schöber test and the evolution time, with the model achieving a R^2^ of 0.697. The stiffness showed a consistent model (R^2^ = 0.630) when the modified Schöber test, the intermalleolar distance, age and the BASFI were combined. The relationship was direct for the intermalleolar distance, age and the BASFI, but inverse for the modified Schöber test. Finally, the decrement was explained by a model that achieved a R^2^ of 0.515, where the variables age, modified Schöber test, height, intermalleolar distance, cervical rotation and tragus to wall distance were inversely related with the elasticity. A similar model pattern was found when relaxation (R^2^ = 0.534) and creep (R^2^ = 0.497) were analyzed, with the modified Schöber test directly related to the outcomes, while the BASFI and the intermalleolar distance were inversely related to the outcomes (Table S2).

## 4. Discussion

In this study, we illustrate the cervical and lumbar myofascial mechanical properties of patients with axSpA, in comparison to healthy controls. Myofascial linear elastic properties (frequency and stiffness) seem to be higher, and viscoelastic properties (relaxation and creep) seem to be lower in patients with axSpA in comparison to healthy controls in both cervical and lumbar regions. All the statistical differences that were detected achieved not only the SEM but also the percentage of MDC identified for all MMPs and both regions, according to our test-retest reliability evaluation. Furthermore, the between-groups differences for tone and stiffness were higher than the differences previously reported in the lumbar region of young adults with chronic low back pain (0.7 Hz and 26.6 N/m, respectively [20,26]) and also of healthy subjects (1.22 Hz and 45.40 N/m, respectively [27]). Therefore, the magnitude of the detected differences for the MMPs can be considered as “real” differences and not due to the measurement method [20], which increases the clinical significance of the results. Furthermore, the abnormalities found are related to different sociodemographic and clinical characteristics, depending on the spinal region. Our study evaluated the five muscle viscoelastic properties that MyotonPro© provides, analyzing not only lumbar muscles which were already analyzed in previous studies [6], but also cervical muscles, which are also affected in axSpA. In addition, we studied these patients with the core set of measurements recommended for them.

Some alterations of spine muscles have been proposed as a possible cause of increased muscle stiffness in axSpA, such as atrophy of the dorsolumbar spine muscles [28,29]. In fact, muscle degeneration has been identified as a cause of kyphotic deformity in axSpA [30]. The histopathology of this degeneration includes type II muscle fiber atrophy and excess endomysial connective tissue in back muscles [31]. Furthermore, paraspinal muscle volume is decreased in axSpA [30]. Our results are in line with previous studies that showed higher lumbar myofascial stiffness as a physical sign in AS patients [6] or in axSpA patients [15,32]. In fact, the current results showed higher tone, stiffness and decrement (linear elastic properties), and lower relaxation and creep (viscoelastic properties) for the axSpA patients than those reported for young asymptomatic subjects [14] and for acute spinal pain patients [15]. Therefore, axSpA increases lumbar and cervical muscle tone, which keeps increasing as the disease progresses [32]. As supported by radiological [33] and histological [34] approaches, the structural changes in paraspinal muscles are in the foreground [7]. Furthermore, Park et al. [35] found differences in cervical muscle frequency and stiffness between patients with cervicogenic headache and healthy individuals, but not in decrement as observed in our patients, which could mean that elasticity does fit the same behavior pattern as other mechanical properties when spinal muscles are assessed. This author also reported a case study [36] of an axSpA patient, analyzing cervical muscles pre- and post-treatment for central and unilateral posteroanterior mobilization. In this study, the treatment was effective in decreasing muscle stiffness.

For cervical muscles, it was identified that there is a consistent relationship between age, decrement and stiffness in healthy women [37], and also in mechanical cervical and lumbar pain [15] in our data of axSpA patients. This relationship was stronger than the relationship between decrement and stiffness with BMI and head posture. We did not find good correlations between the same measurements in cervical and lumbar muscles. For cervical muscles, the best correlated measurement was decrement, and for lumbar muscles it was frequency and stiffness. These could be due to the fact that the myotonometry features of these two muscle groups are slightly different: they show higher values in axSpA, but correlations with other variables, for example age, are different. Another explanation for these different responses of the cervical and lumbar muscles could be related to their histophysiological characteristics. Although both cervical and lumbar muscles have a predominance of type I fibers, indicative of their postural character [38,39], it is known that there are differences between the muscles of different anatomical regions at the level of component molecules that reflect their differences in the development of active voltage, passive voltage and mechanosensitive signaling [40].

As commented, we found a high correlation of lumbar frequency, stiffness, relaxation, creep and cervical decrement, with age and metrology variables. However, only lumbar myotonometric outcomes were related to functional status, activity of disease, structural damage or quality of life. This pattern found a pooled reflex in the linear regression analysis of our study that produced moderate to good fitted models (0.328 < R^2^ < 0.697) for estimation of cervical and lumbar MMPs, mainly in the lumbar region. Again, the most significant variables introduced in the models were age, probably associated with the loss of skeletal muscle mass that occurs with advancing time [15], and conventional metrology. In addition to these, activity of the disease, as occurred in cervical decrement and lumbar frequency, and function, being the case of lumbar relaxation and creep, were included in the models. That is, an alteration of the mechanical characteristics of the muscle was accompanied by an extensive alteration of clinical and psychophysiological features of axSpA, with high intensity in the lumbar region. These associations between paraspinal muscle deterioration at lumbar level and both activity of the disease and functionality in axSpa have been previously described, suggesting that paraspinal muscles increase both spinal motor activity and radiological progression deterioration [7,33]. Furthermore, the relationship between paraspinal muscle alterations with pain and a worse prognosis has also been described in chronic low back pain [41], probably due to the continuous activity of paraspinal muscles in the lumbopelvic biomechanics. These approaches increase the relevance of assessing the MMPs as a possible marker of the axSpA status and progression, as proposed in low back pain [15].

It is interesting to link our findings with recent theories, suggesting that structural biomechanical processes lead to tissue reactions and might possibly precede initiation of other pathophysiological pathways [4]. One of these processes is the mechanotransduction associated with myofascial tissue. Thus, the physiological changes in connective tissues after the application of a mechanical stimulus can become aberrant when some factors are present, such as excessive mechanical stress, inflammation or pain [42]. More research is necessary to understand the pathophysiology of mechanotransduction in axSpA, but it is well described in other musculoskeletal disorders as the basis of some treatment approaches [43]. This relationship might improve current understanding of the initiation and progression of axSpa and deserves further research.

The main limitation of our study is inherent to the case-control studies, as even if we described a correlation between biomechanical abnormalities and certain clinical parameters, we could not establish causation, namely what is the case and what is the effect. Furthermore, the current results could only be extrapolated to similar samples because it is well known that spinal rheumatism shows heterogenous clinical patterns [7]. Some authors use surface electromyography (sEMG) to ensure that the muscle is at rest when tone is measured [44], but recent research has successfully used myotonometry of spinal muscles in a clinical setting without EMG as a control mechanism [15].

## 5. Conclusions

In conclusion, cervical and lumbar paraspinal muscle tone, stiffness (linear elastic properties) and lower relaxation and creep (viscoelastic properties), evaluated using a myotonomotry device, was altered in axSpA when compared to healthy individuals. These mechanical characteristics were related to age, functional status, activity of disease, structural damage and quality of life, in a higher intensity for lumbar region. The physiopathological and clinical implications of these abnormalities merit further research.

## Figures and Tables

**Table 1:** Demographic characteristics of the sample.

	axSpA (*n* = 34)	Control (*n* = 34)	*p*
Age (years)	46.21 (8.53)	43.97 (8.49)	NS
Sex. female (%)	10 (29.4%)	10 (29.4%)	
Weight (Kg)	74.58 (7.15)	74.79 (12.85)	NS
Height (cm)	171.20 (9.42)	173.09 (12.91)	NS
BMI (Kg/m^2^)	25.39 (3.46)	25.01 (4.13)	NS
Evolution time (years)	16.70 (14.19)	-	
BASMI	3.40 (1.97)	1.66 (0.64)	***
BASFI	3.12 (2.71)	-	
BASDAI	3.90 (2.40)	-	
mSASSS	12.12 (17.07)	-	
PCS-12	44.05 (9.95)	-	
MCS-12	50.98 (9.31)	-	
Lateral flexion (cm)	13.07 (9.30)	22.52 (18.35)	**
Tragus to wall distance (cm)	13.07 (5.19)	11.15 (1.15)	*
Schöber test (cm)	4.72 (1.98)	5.33 (1.03)	NS
Intermalleolar distance (cm)	93.97 (19.48)	119.22 (15.06)	***
Cervical rotation (°)	55.33 (21.65)	73.29 (14.43)	***

BMI: body mass index; BASMI, Bath Ankylosing Spondylitis Metrology Index; BASFI, Bath Ankylosing Spondylitis Functional index; ASAS-HI, ASAS Health Index; BASDAI, Bath Ankylosing Spondylitis Disease Activity Index; BAS-G, Bath Ankylosing Spondylitis Global Index; mSASSS; PCS-12, physical component of the 12-item Short-Form Health Survey (SF-12); MCS-12, mental component of the 12-item Short-Form Health Survey (SF-12). * *p* < 0.05, ** *p* < 0.01, *** *p* < 0.001, NS not significant differences.

**Table 2:** Results of muscle tone measurements in axSpA patients (*n* = 34), healthy controls (*n* = 34) and significance of differences between both groups.

	Cervical			Lumbar		
	axSpA	Control	*p*	axSpA	Control	*p*
Frequency (Hz)	18.02 (1.49)	15.68 (1.82)	***	17.95 (4.46)	15.93 (1.89)	*
Stiffness (N/m)	349.60 (44.90)	273.27 (55.14)	***	393.57 (152.24)	320.69 (64.74)	*
Decrement	1.35 (0.24)	1.39 (0.14)	NS	1.40 (0.32)	1.43 (0.33)	NS
Relaxation (ms)	15.07 (1.60)	19.41 (4.17)	***	14.79 (4.31)	17.13 (2.92)	**
Creep	0.94 (0.10)	1.18 (0.24)	***	0.92 (0.23)	1.06 (0.15)	**

* *p* < 0.05, ** *p* < 0.01, *** *p* << 0.001, NS: not significant differences.

**Table 3:** Bivariate correlations (Pearson) between muscle tone measurements and other measured variables in axSpA patients.

	Cervical					Lumbar				
	Frequency	Stiffness	Decrement	Relaxation	Creep	Frequency	Stiffness	Decrement	Relaxation	Creep
Age	0.459 **	0.346 *	0.655 **	−0.148	0.003	0.515 **	0.503 **	0.257	−0.426 *	−0.404 *
Height	−0.308	−0.316	−0.179	0.207	0.168	0.104	0.051	0.108	−0.053	−0.024
Weight	−0.142	0.022	0.272	0.070	0.136	0.084	0.068	−0.033	0.049	0.074
BMI	0.022	0.228	0.426 *	−0.051	0.052	0.040	0.051	−0.092	0.090	0.102
Evolution time	−0.080	−0.006	0.120	0.009	0.016	−0.226	−0.192	−0.179	0.227	0.217
Lateral flexion	−0.213	−0.148	−0.374 *	0.120	0.062	−0.487 **	−0.506 **	−0.096	0.526 **	0.460 **
Tragus-wall	0.051	0.057	0.500 **	0.114	0.244	0.682 **	0.693 **	−0.029	−0.579 **	−0.574 **
Schöber test	−0.023	0.044	−0.467 **	−0.101	−0.173	−0.670 **	−0.697 **	0.174	0.618 **	0.608 **
Intermalleolar distance	−0.142	−0.050	−0.362 *	0.000	−0.055	−0.146	−0.232	−0.149	0.207	0.179
Cervical rotation	−0.047	0.024	−0.562 **	−0.199	−0.324	−0.489 **	−0.493 **	0.018	0.317	0.303
Chest expansion	−0.051	−0.040	−0.550 **	−0.071	−0.153	−0.315	−0.339	−0.004	0.230	0.204
BASMI	0.052	−0.010	0.536 **	0.138	0.239	0.613 **	0.646 **	−0.071	−0.559 **	−0.557 **
BASDAI	0.175	0.036	−0.037	0.080	0.121	0.370 *	0.326	0.219	−0.284	−0.286
BASFI	0.042	−0.014	0.294	0.170	0.251	0.508 **	0.563 **	0.092	−0.522 **	−0.532 **
Pain	0.138	0.035	−0.016	0.075	0.112	0.425 *	0.381 *	0.294	−0.353 *	−0.346 *
mSASSS	0.241	0.183	0.676 **	−0.044	0.082	0.774 **	0.728 **	−0.327	−0.489	−0.534 *
PCS-12	−0.070	0.126	0.152	−0.158	−0.209	−0.308	−0.208	−0.198	0.285	0.274
MCS-12	−0.164	0.020	0.091	−0.033	−0.098	−0.444	−0.342	−0.138	0.415	0.414

* *p* < 0.05, ** *p* < 0.01.

## Data Availability

The data presented in this study are available upon reasonable request from the corresponding author.

## Supplementary Materials

The following are available online at www.mdpi.com/2075-4418/11/9/1662/s1, Table S1: Summary of the stepwise regression analyses to determine predictors of cervical muscles frequency, stiffness and decrement; Table S2: Summary of the stepwise regression analyses to determine predictors of lumbar muscles frequency, stiffness, decrement, relaxation and creep.

## References

[1] Dougados M., Baeten D. (2011). Spondyloarthritis. Lancet.

[2] Ronneberger M., Schett G. (2011). Pathophysiology of spondyloarthritis. Curr. Rheumatol. Rep..

[3] de Koning A., Schoones J.W., van der Heijde D., van Gaalen F.A. (2018). Pathophysiology of axial spondyloarthritis: Consensus and controversies. Eur. J. Clin. Invest..

[4] Masi A.T., Nair K., Andonian B.J., Prus K.M., Kelly J., Sanchez J.R., Henderson J. (2011). Integrative Structural Biomechanical Concepts of Ankylosing Spondylitis. Arthritis.

[5] Masi A.T., Hannon J.C. (2008). Human resting muscle tone (HRMT): Narrative introduction and modern concepts. J. Bodyw. Mov. Ther..

[6] Andonian B.J., Masi A.T., Aldag J.C., Barry A.J., Coates B.A., Emrich K., Henderson J., Kelly J., Nair K. (2015). Greater Resting Lumbar Extensor Myofascial Stiffness in Younger Ankylosing Spondylitis Patients Than Age-Comparable Healthy Volunteers Quantified by Myotonometry. Arch. Phys. Med. Rehabil..

[7] Ozturk E.C., Yagci I. (2021). The structural, functional and electrophysiological assessment of paraspinal musculature of patients with ankylosing spondylitis and non-radiographic axial spondyloarthropathy. Rheumatol. Int..

[8] Aranda-Valera I.C., Garrido-Castro J.L., Martínez-Galisteo A., Peña-Amaro J., González-Navas C., Cuesta-Vargas A., Jiménez-Reina L., Collantes-Estévez E., López-Medina C. (2021). Patients with axial spondyloarthritis show an altered flexion/relaxation phenomenon. Diagnostics.

[9] Valido A., Crespo C.L., Pimentel-Santos F.M. (2019). Muscle Evaluation in Axial Spondyloarthritis—The Evidence for Sarcopenia. Front. Med..

[10] Meerits T., Bacchieri S., Pääsuke M., Ereline J., Cicchella A., Gapeyeva H. (2014). Acute effect of static and dynamic stretching on tone and elasticity of hamstring muscle and on vertical jump performance in track-and-field athletes. Acta Kinesiol. Univ. Tartu..

[11] Chuang L.L., Wu C.Y., Lin K.C. (2012). Reliability, validity, and responsiveness of myotonometric measurement of muscle tone, elasticity, and stiffness in patients with stroke. Arch. Phys. Med. Rehabil..

[12] Dougherty J., Schaefer E., Nair K., Kelly J., Masi A. Repeatability, reproducibility, and calibration of the myotonpro^®^ on tissue mimicking phantoms. Proceedings of the ASME 2013 Summer Bioengineering Conference.

[13] Marusiak J., Kisiel-Sajewicz K., Jaskólska A., Jaskólski A. (2010). Higher Muscle Passive Stiffness in Parkinson’s Disease Patients Than in Controls Measured by Myotonometry. Arch. Phys. Med. Rehabil..

[14] Kelly J.P., Koppenhaver S.L., Michener L.A., Proulx L., Bisagni F., Cleland J.A. (2018). Characterization of tissue stiffness of the infraspinatus, erector spinae, and gastrocnemius muscle using ultrasound shear wave elastography and superficial mechanical deformation. J. Electromyogr. Kinesiol..

[15] Alcaraz-Clariana S., García-Luque L., Garrido-castro J.L., Carmona-Pérez C., Rodrigues-de-souza D.P. (2021). Paravertebral Muscle Mechanical Properties and Spinal Range of Motion in Patients with Acute Neck or Low Back Pain: A Case-Control Study. Diagnostics.

[16] Myoton A.S. (2012). MyotonPro User Manual.

[17] Pruyn E.C., Watsford M., Murphy A. (2014). The relationship between lower-body stiffness and dynamic performance. Appl. Physiol. Nutr. Metab..

[18] Pruyn E.C., Watsford M.L., Murphy A.J. (2016). Validity and reliability of three methods of stiffness assessment. J. Sport Health Sci..

[19] Leonard C.T., Brown J.S., Price T.R., Queen S.A., Mikhailenok E.L. (2004). Comparison of surface electromyography and myotonometric measurements during voluntary isometric contractions. J. Electromyogr. Kinesiol..

[20] Hu X., Lei D., Li L., Leng Y., Yu Q., Wei X., Lo W.L.A. (2018). Quantifying paraspinal muscle tone and stiffness in young adults with chronic low back pain: A reliability study. Sci. Rep..

[21] Rudwaleit M., van der Heijde D., Landewé R., Listing J., Akkoc N., Brandt J., Braun J., Chou C.T., Collantes-Estevez E., Dougados M. (2009). The development of Assessment of Spondylo Arthritis international Society classification criteria for axial spondyloarthritis (part II): Validation and final selection. Ann. Rheum. Dis..

[22] Sieper J., Rudwaleit M., Baraliakos X., Brandt J., Braun J., Burgos-Vargas R., Dougados M., Hermann K.G., Landewe R., Maksymowych W. (2009). The Assessment of SpondyloArthritis international Society (ASAS) handbook: A guide to assess spondyloarthritis. Ann. Rheum. Dis..

[23] Ware J.E., Kosinski M., Keller S.D. (1996). A 12-Item Short-Form Health Survey of Scales and Preliminary Construction Tests of Reliability and Validity. Med. Care.

[24] Resnick B., Parker B., Resnick A. (2001). Simplified scoring and psychometrics of the revised 12-item Short-Form Health Survey. Outcomes Manag. Nurs. Pract..

[25] Akoglu H. (2018). User’s guide to correlation coefficients. Turk. J. Emerg. Med..

[26] Wai Leung Ambrose L., Qiuhua Y., Yurong M., Wenfeng L., Chengpeng H., Le L. (2019). Lumbar muscles biomechanical characteristics in young people with chronic spinal pain. BMC Musculoskelet. Disord..

[27] Lohr C., Braumann K.M., Reer R., Schroeder J., Schmidt T. (2018). Reliability of tensiomyography and myotonometry in detecting mechanical and contractile characteristics of the lumbar erector spinae in healthy volunteers. Eur. J. Appl. Physiol..

[28] Forestier J., Jacqueline F., Rotes-Querol J. (1951). La Spondylarthrite Ankylosante: Clinique, Radiologie Anatomie Pathologique, Traitement.

[29] Masi A.T., Sierakowski S., Kim J.M. (2005). Jacques Forestier’s vanished bowstring sign in ankylosing spondylitis: A call to test its validity and possible relation to spinal myofascial hypertonicity. Clin. Exp. Rheumatol..

[30] Bok D.H., Kim J., Kim T.H. (2017). Comparison of MRI-defined back muscles volume between patients with ankylosing spondylitis and control patients with chronic back pain: Age and spinopelvic alignment matched study. Eur. Spine J..

[31] Cooper R.G., Freemont A.J., Fitzmaurice R., Alani S.M., Jayson M.I. (1991). V Paraspinal muscle fibrosis: A specific pathological component in ankylosing spondylitis. Ann. Rheum. Dis..

[32] White A., Abbott H., Masi A.T., Henderson J., Nair K. (2018). Biomechanical properties of low back myofascial tissue in younger adult ankylosing spondylitis patients and matched healthy control subjects. Clin. Biomech..

[33] Akgul O., Gulkesen A., Akgol G., Ozgocmen S. (2013). MR-defined fat infiltration of the lumbar paravertebral muscles differs between non-radiographic axial spondyloarthritis and established ankylosing spondylitis. Mod. Rheumatol..

[34] Zhang Y., Xu H., Hu X., Zhang C., Chu T., Zhou Y. (2016). Histopathological changes in supraspinous ligaments, ligamentum flava and paraspinal muscle tissues of patients with ankylosing spondylitis. Int. J. Reum. Dis..

[35] Park S.K., Yang D.J., Uhm Y.H., Yoon J.H., Kim J.H. (2018). Effects of extracorporeal shock wave therapy on upper extremity muscle tone in chronic stroke patients. J. Phys. Ther. Sci..

[36] Park S.E., Kim B.K., Lee S.B., Choi W.S., Yeum D.M. (2017). Effects of central and unilateral posteroanterior mobilization on cervical lordosis, muscle stiffness and ROM in patient with ankylosing spondylitis: Case study. J. Phys. Ther. Sci..

[37] Kocur P., Tomczak M., Wiernicka M., Goliwąs M., Lewandowski J., Łochyński D. (2019). Relationship between age, BMI, head posture and superficial neck muscle stiffness and elasticity in adult women. Sci. Rep..

[38] Cornwall J., Kennedy E. (2015). Fiber types of the anterior and lateral cervical muscles in elderly males. Eur. Spine J..

[39] Regev G.J., Kim C.W., Thacker B.E., Tomiya A., Garfin S.R., Ward S.R., Lieber R.L. (2010). Regional Myosin heavy chain distribution in selected paraspinal muscles. Spine.

[40] Tirrell T.F., Cook M.S., Carr J.A., Lin E., Ward S.R., Lieber R.L. (2012). Human skeletal muscle biochemical diversity. J. Exp. Biol..

[41] Ranger T.A., Cicuttini F.M., Jensen T.S., Peiris W.L., Hussain S.M., Fairley J., Urquhart D.M. (2017). Are the size and composition of the paraspinal muscles associated with low back pain? A systematic review. Spine J..

[42] Ingber D.E. (2003). Mechanobiology and diseases of mechanotransduction. Ann. Med..

[43] Camargo P.R., Alburquerque-Sendín F., Salvini T.F. (2014). Eccentric training as a new approach for rotator cufftendinopathy: Review and perspectives. World J. Orthop..

[44] Nair K., Masi A.T., Andonian B.J., Barry A.J., Coates B.A., Dougherty J., Schaefer E., Henderson J., Kelly J. (2016). Stiffness of resting lumbar myofascia in healthy young subjects quantified using a handheld myotonometer and concurrently with surface electromyography monitoring. J. Bodyw. Mov. Ther..

