# Supplementary material for: Mechanical Properties of Lumbar and Cervical Paravertebral Muscles in Patients with Axial Spondyloarthritis: A Case-Control Study
Source: Diagnostics (Basel). 2021 Sep 10;11(9):1662. doi: 10.3390/diagnostics11091662 (PMC8472659; doi:10.3390/diagnostics11091662)
Supplement: Supplementary file 1 [file diagnostics-11-01662-s001.zip › diagnostics-1324557-supplementary.pdf]

**Table S1.** Summary of the Stepwise Regression Analyses to determine Predictors of cervical muscles frequency, stiffness and decrement.

| | Predictor Outcome | B | SE B | 95% CI | *B* | t | P |
|---|---|---|---|---|---|---|---|
| Frequency | Step 1 (adjusted $R^2$ = .298) | | | | | | |
| | Age | .074 | .015 | .045, .104 | .556 | 5.100 | <.001 |
| | Step 2 (adjusted $R^2$ = .385) | | | | | | |
| | Age | .102 | .016 | .069, .135 | .764 | 6.212 | <.001 |
| | Tragus-wall | -.102 | .034 | -.170, -.035 | -.373 | -3.030 | .004 |
| | Step 3 (adjusted $R^2$ = .422) | | | | | | |
| | Age | .099 | .016 | .067, .131 | .743 | 6.208 | <.001 |
| | Tragus-wall | -.136 | .036 | -.209, -.063 | -.497 | -3.753 | <.001 |
| | Lateral flexion | -.040 | .019 | -.077, -.003 | -.254 | -2.159 | .035 |
| Stiffness | Step 1 (adjusted $R^2$ = .264) | | | | | | |
| | Age | 2.048 | .435 | 1.176, 2.919 | .525 | 4.703 | <.001 |
| | Step 2 (adjusted $R^2$ = .328) | | | | | | |
| | Age | 2.824 | .514 | 1.794, 3.853 | .725 | 5.491 | <.001 |
| | Cervical rotation | .692 | .270 | .152, 1.233 | .338 | 2.565 | .013 |
| Decrement | Step 1 (adjusted $R^2$ = .423) | | | | | | |
| | Age | .015 | .002 | .010, .019 | .658 | 6.646 | <.001 |
| | Step 2 (adjusted $R^2$ = .523) | | | | | | |
| | Age | .017 | .002 | .013, .022 | .783 | 8.131 | <.001 |
| | BASDAI | .036 | .010 | .056, .016 | .350 | 3.637 | .001 |
| | Step 3 (adjusted $R^2$ = .615) | | | | | | |
| | Age | .013 | .002 | .009, .018 | .595 | 5.970 | <.001 |
| | BASDAI | .048 | .009 | .067, .029 | .463 | 2.729 | <.001 |
| | BASMI | .050 | .013 | .024, .076 | .399 | 3.831 | <.001 |

SE: standard error; CI: confidence interval.

**Table S2.** Summary of the Stepwise Regression Analyses to determine Predictors of lumbar muscles frequency, stiffness, decrement, relaxation and creep.

| | Predictor Outcome | B | SE B | 95% CI | *B* | t | P |
|---|---|---|---|---|---|---|---|
| Frequency | Step 1 (adjusted $R^2 = .504$) | | | | | | |
| | Intermalleolar distance | .064 | .027 | .010, .118 | .244 | 2.367 | .021 |
| | Step 2 (adjusted $R^2 = .578$) | | | | | | |
| | Intermalleolar distance | .092 | .026 | .039, .144 | .351 | 3.504 | .001 |
| | Schöber test | -1.149 | .345 | -1.841, -.457 | -.504 | -3.328 | .002 |
| | Step 3 (adjusted $R^2 = .643$) | | | | | | |
| | Intermalleolar distance | .112 | .025 | .063, .162 | .429 | 4.556 | <.001 |
| | Schöber test | -1.672 | .218 | -2.109, -1.235 | -.734 | -7.666 | <.001 |
| | Age | .168 | .036 | .008, .031 | .403 | 4.605 | <.001 |
| | Step 4 (adjusted $R^2 = .679$) | | | | | | |
| | Intermalleolar distance | .126 | .024 | .078, .174 | .482 | 5.278 | <.001 |
| | Schöber test | -1.679 | .207 | -2.093, -1.265 | -.736 | -8.123 | <.001 |
| | Age | .143 | .036 | .071, .214 | .343 | 3.994 | <.001 |
| | BASDAI | .429 | .158 | .113, -.745 | .221 | 2.719 | .009 |
| | Step 5 (adjusted $R^2 = .697$) | | | | | | |
| | Intermalleolar distance | .127 | .023 | .080, .173 | .485 | 5.460 | <.001 |
| | Schöber test | -1.643 | .202 | -2.047, -1.239 | -.721 | -8.146 | <.001 |
| | Age | .144 | .035 | .074, .214 | .346 | 4.146 | <.001 |
| | BASDAI | .439 | .153 | .131, .746 | .226 | 2.857 | .006 |
| | Evolution time | -.088 | .043 | -.175, -.002 | -.148 | -2.049 | .045 |
| Stiffness | Step 1 (adjusted $R^2 = .524$) | | | | | | |
| | Schöber test | -29.509 | 11.824 | -53.187, -5.832 | -.380 | -2.496 | .015 |
| | Step 2 (adjusted $R^2 = .557$) | | | | | | |
| | Schöber test | -38.394 | 12.055 | -62.543, -14.245 | -.495 | -3.185 | .002 |
| | Intermalleolar distance | 2.090 | .915 | .257, 3.923 | .235 | 2.284 | .026 |
| | Step 3 (adjusted $R^2 = .604$) | | | | | | |
| | Schöber test | -42.738 | 11.497 | -65.778, -19.698 | -.551 | -3.717 | <.001 |
| | Intermalleolar distance | 2.609 | .885 | .837, 4.382 | .293 | 2.950 | .005 |
| | Age | 3.986 | 1.434 | 1.113, 6.859 | .281 | 2.780 | .007 |
| | Step 4 (adjusted $R^2 = .630$) | | | | | | |
| | Schöber test | -49.516 | 7.924 | -65.395, -33.637 | -.638 | -6.249 | <.001 |
| | Intermalleolar distance | 3.333 | .893 | 1.543, 5.123 | .374 | 3.732 | <.001 |
| | Age | 3.785 | 1.336 | 1.108, 6.461 | .267 | 2.834 | .006 |
| | BASFI | 15.631 | 6.227 | 3.151, 28.111 | .269 | 2.510 | 0.015 |

| | | | | | | | |
|---|---|---|---|---|---|---|---|
| Decrement | Step 1 (adjusted $R^2$ = .050) | | | | | | |
| | Age | .008 | .004 | .000, .015 | .255 | 2.007 | .049 |
| | Step 2 (adjusted $R^2$ = .153) | | | | | | |
| | Age | .012 | .004 | .004, .020 | .412 | 3.124 | .004 |
| | Schöber test | .062 | .022 | .018, .105 | .377 | 2.858 | .006 |
| | Step 3 (adjusted $R^2$ = .285) | | | | | | |
| | Age | .018 | .004 | .010, .025 | .589 | 4.468 | <.001 |
| | Schöber test | .082 | .021 | .040, .123 | .499 | 3.951 | <.001 |
| | Height | .019 | .006 | .008, .031 | .410 | 3.396 | .001 |
| | Step 4 (adjusted $R^2$ = .367) | | | | | | |
| | Age | .016 | .004 | .009, .024 | .545 | 4.362 | <.001 |
| | Schöber test | .112 | .022 | .068, .156 | .685 | 5.060 | <.001 |
| | Height | .022 | .005 | .011, .033 | .474 | 4.091 | <.001 |
| | Intermalleolar distance | -.007 | .002 | -.012, -.002 | -.367 | -2.870 | .006 |
| | Step 5 (adjusted $R^2$ = .470) | | | | | | |
| | Age | .023 | .004 | .015, .031 | .770 | 5.831 | <.001 |
| | Schöber test | .099 | .021 | .058, .141 | .606 | 4.814 | <.001 |
| | Height | .027 | .005 | .017, .038 | .580 | 5.249 | <.001 |
| | Intermalleolar distance | -.010 | .002 | -.015, -.005 | -.523 | -4.163 | <.001 |
| | Cervical rotation | .008 | .002 | .003, .012 | .483 | 3.409 | .001 |
| Relaxation | Step 1 (adjusted $R^2$ = .367) | | | | | | |
| | Schöber test | 1.313 | .221 | .870, 1.756 | .614 | 5.930 | <.001 |
| | Step 2 (adjusted $R^2$ = .403) | | | | | | |
| | Schöber test | 1.008 | .258 | .492, 1.525 | .472 | 3.908 | <.001 |
| | BASFI | -.413 | .193 | -.800, -.026 | -.258 | -2.135 | .037 |
| | Step 3 (adjusted $R^2$ = .534) | | | | | | |
| | Schöber test | 1.163 | .245 | .672, 1.654 | .544 | 4.745 | <.001 |
| | BASFI | -1.092 | .248 | -1.590, -.594 | -.682 | -4.398 | <.001 |
| | Intermalleolar distance | -.069 | .028 | -.126, -.013 | -.283 | -2.473 | .017 |
| Creep | Step 1 (adjusted $R^2$ = .355) | | | | | | |
| | Schöber test | .070 | .012 | .046, .094 | .605 | 5.781 | <.001 |
| | Step 2 (adjusted $R^2$ = .398) | | | | | | |
| | Schöber test | .052 | .014 | .024, .080 | .452 | 3.726 | <.001 |
| | BASFI | -.024 | .011 | -.045, -.003 | -.277 | -2.283 | .026 |
| | Step 3 (adjusted $R^2$ = .497) | | | | | | |
| | Schöber test | .068 | .014 | .041, .096 | .591 | 5.017 | <.001 |
| | BASFI | -.036 | .010 | -.057, -.016 | -.418 | -3.544 | .001 |
| | Intermalleolar distance | -.005 | .002 | -.009, .002 | -.406 | -3.492 | .001 |

SE: standard error; CI: confidence interval.
